# *LINE-1* hypomethylation in human hepatocellular carcinomas correlates with shorter overall survival and CIMP phenotype

**DOI:** 10.1371/journal.pone.0216374

**Published:** 2019-05-06

**Authors:** Sumadi Lukman Anwar, Britta Hasemeier, Elisa Schipper, Arndt Vogel, Hans Kreipe, Ulrich Lehmann

**Affiliations:** 1 Division of Surgical Oncology Department of Surgery, Faculty of Medicine Public Health and Nursing, Universitas Gadjah Mada, Yogyakarta, Indonesia; 2 Institute of Pathology, Medizinische Hochschule Hannover, Hannover, Germany; 3 Department of Gastroenterology, Hepatology and Endocrinology, Medizinische Hochschule Hannover, Hannover, Germany; University of Navarra School of Medicine and Center for Applied Medical Research (CIMA), SPAIN

## Abstract

Reactivation of interspersed repetitive sequences due to loss of methylation is associated with genomic instability, one of the hallmarks of cancer cells. *LINE-1* hypomethylation is a surrogate marker for global methylation loss and is potentially a new diagnostic and prognostic biomarker in tumors. However, the correlation of *LINE-1* hypomethylation with clinicopathological parameters and the CpG island methylator phenotype (CIMP) in patients with liver tumors is not yet well defined, particularly in Caucasians who show quite low rates of HCV/HBV infection and a higher incidence of liver steatosis. Therefore, quantitative DNA methylation analysis of *LINE-1*, *RASSF1A*, and *CCND2* using pyrosequencing was performed in human hepatocellular carcinomas (HCC, n = 40), hepatocellular adenoma (HCA, n = 10), focal nodular hyperplasia (FNH, n = 5), and corresponding peritumoral liver tissues as well as healthy liver tissues (n = 5) from Caucasian patients. Methylation results were correlated with histopathological findings and clinical data. We found loss of *LINE-1* DNA methylation only in HCC. It correlated significantly with poor survival (log rank test, *p* = 0.007). An inverse correlation was found for *LINE-1* and *RASSF1A* DNA methylation levels (r^2^ = -0.47, *p* = 0.002). *LINE-1* hypomethylation correlated with concurrent *RASSF1*/*CCND2* hypermethylation (Fisher’s exact test, *p* = 0.02). Both *LINE-1* hypomethylation and *RASSF1A*/*CCND2* hypermethylation were not found in benign hepatocellular tumors (HCA and FNH). Our results show that *LINE-1* hypomethylation and *RASSF1A*/*CCND2* hypermethylation are epigenetic aberrations specific for the process of malignant liver transformation. In addition, *LINE-1* hypomethylation might serve as a future predictive biomarker to identify HCC patients with unfavorable overall survival.

## Introduction

Long Interspersed Nucleotide Element 1 (*LINE-1*) is a major repetitive DNA sequence comprising up to ~17% of the human genome [1, 2]. There are 3 classes of repetitive sequences: terminal repeats, tandem repeats (satellite DNA, minisatellites, and microsatellites including centromeres and telomeres), and interspersed repeats (transposons and retrotransposons including *LINE-1* and *Alu*) [2, 3]. *LINE-1* is considered as the most active mobile element in mediating retrotransposition [3, 4]. Epigenetic mechanisms, in particular DNA methylation, maintain the repetitive elements including *LINE-1* in an inactive state [3, 5]. Reactivation of *LINE-1* protein produces more copies of DNA elements which results in a higher chance of pathogenic gene insertions and gene translocations thereby contributing to genomic instability [6], chromosomal breakage [7], and oncogenic activation.

Human hepatocellular carcinoma (HCC) is the fifth most frequently diagnosed cancer with a total incidence of around 840,000 cases worldwide [8]. Although there have been recent advances in the diagnosis and treatment, the mortality rate of HCC is relatively high, reaching 780,000 cases per year [8]. This indicates that new strategies are required to improve clinical management of HCC including development of novel diagnostic and prognostic biomarkers. Liver carcinogenesis is a multistep process involving diverse alterations of both genetics and epigenetics during the disease development and progression [9]. Among other epigenetic alterations, DNA methylation is the longest and best studied in which cancer cells often show promoter gene-specific hypermethylation [10]. In HCC, we have previously reported and summarized specific gene promoter hypermethylation in protein-encoding genes [11], microRNA genes [12–14], and imprinted genes [15–17].

The majority of CpG dinucleotides in mammals are methylated except those contained within CpG islands encompassing active gene promoters [10]. It has been shown that DNA methylation is able to initiate a cascade of biological process to stably silence gene expression [18]. In cancer, gene-specific hypermethylation is frequently accompanied by global loss of DNA methylation [9, 10]. In healthy cells, repetitive elements that comprise two thirds of the human genome are tightly regulated and maintained in inactive states through DNA methylation as a natural defense mechanism against autonomic replication, transposition, and insertion [3]. Global loss of methylation in cancer cells primarily affects repetitive elements thereby activating the repeats to start transposition and induce genomic instability [6]. Several studies have shown that *LINE-1* DNA methylation reflects the levels of global DNA methylation [19]. *LINE-1* hypomethylation has been reported in some gastrointestinal cancers including colorectal cancer [20], esophageal cancer [21], gastric cancer [22] and correlated with poorer clinical outcome. Although correlation of *LINE-1* hypomethylation and unfavorable HCC outcome has been previously reported in patient cohorts from East Asia [23–25], there is no previous report including benign liver tumors and the comparison with adjacent healthy liver tissues.

In the present study we analyzed *LINE-1*, *RASSF1A*, and *CCND2* DNA methylation levels in HCC, HCA, FNH, corresponding adjacent liver tissues, and healthy liver tissues in Caucasian individuals. We found a correlation between *LINE-1* hypomethylation with worse overall survival, and concurrent *RASSF1*/*CCND2* hypermethylation (CIMP phenotype).

## Results

### Loss of *LINE-1* DNA methylation in HCC cell lines and HCC primary tissues

**Fig 1:**
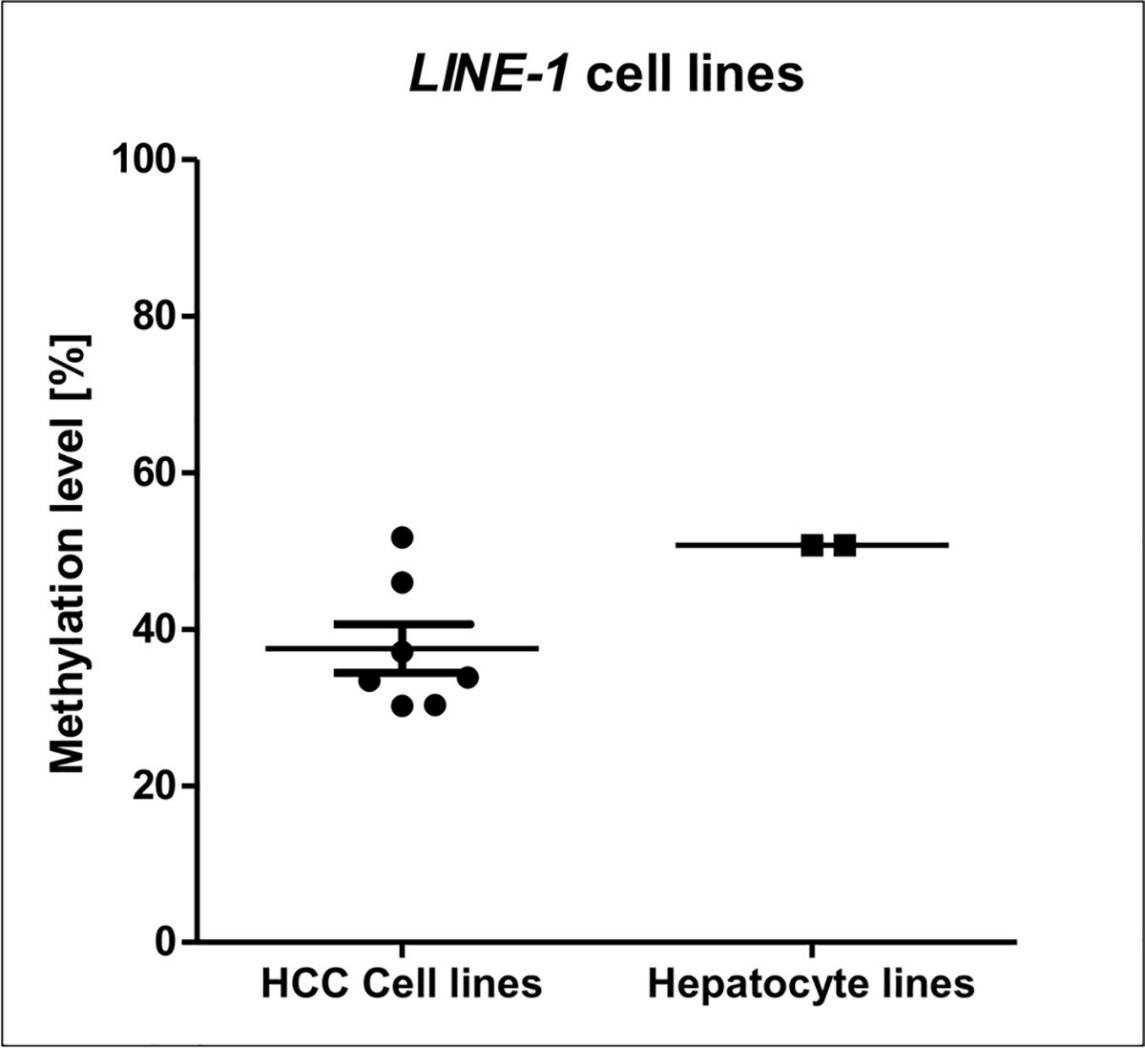
*LINE-1* DNA methylation levels in HCC cell lines and hepatocyte cell lines. Seven HCC lines showed significant lower DNA methylation levels than hepatocytes lines (mean methylation37.52 ± 3.12 vs. 50.73 ± 0.02 respectively, *p* = 0.005). For DNA methylation levels of individual CpG sites see S2 Table.

**Fig 2:**
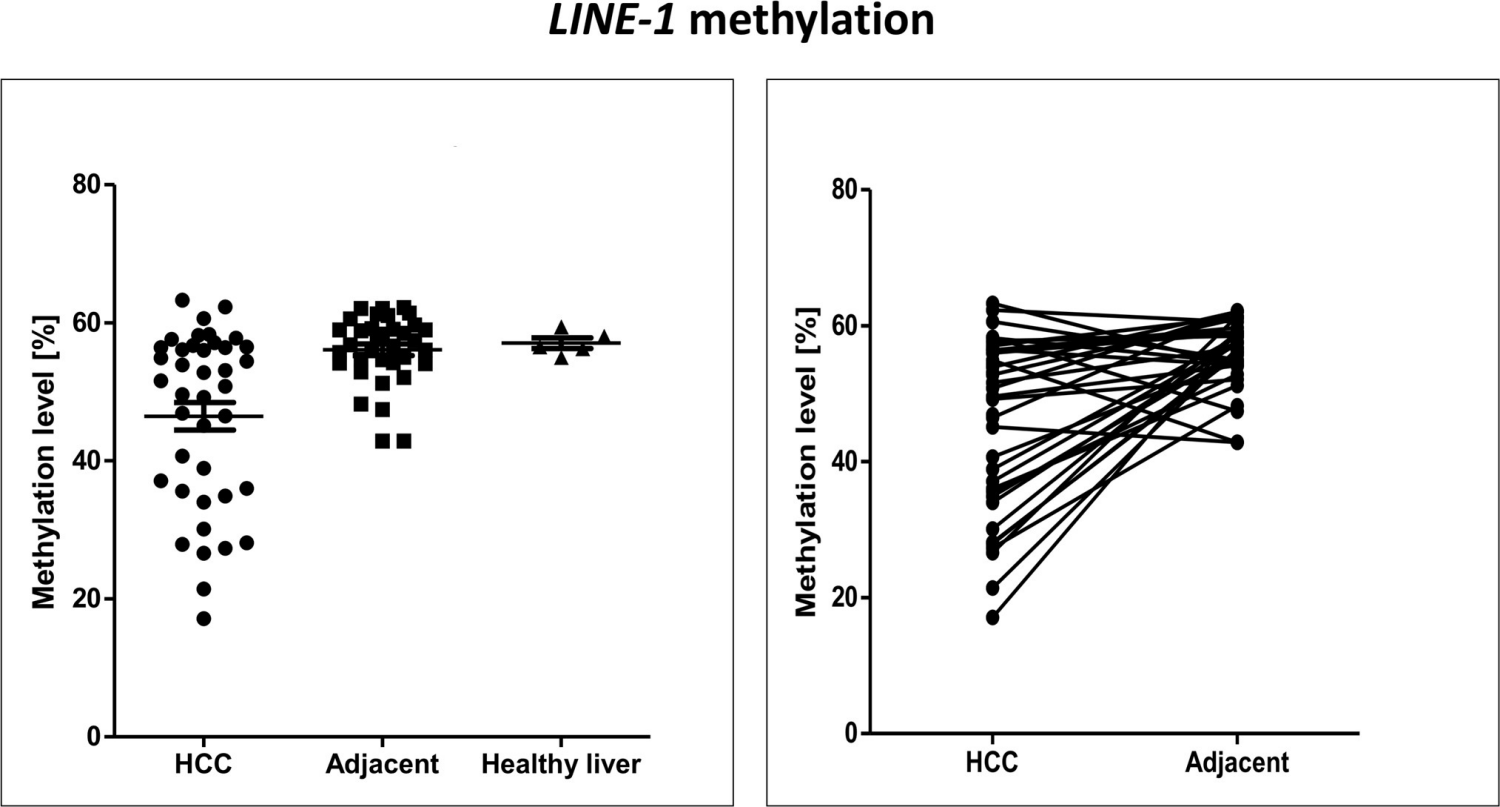
*LINE-1* DNA methylation in HCC, adjacent peritumoral tissues, and healthy liver. Frequent loss of *LINE-1* DNA methylation was observed in HCC primary tissues. Means of DNA methylation were 46.5, 56.1, and 57.1 in HCC, peritumoral, and healthy liver tissues respectively. *LINE-1* DNA methylation levels were significantly lower in the HCC primary tissues compared to the adjacent peritumoral tissues and significant difference was not observed between peritumoral tissues and healthy liver tissues. For DNA methylation levels of individual CpG sites see S3 Table.

We measured *LINE-1* DNA methylation levels in HCC cell lines (n = 7) and hepatocyte lines (n = 2). HCC cell lines showed significant lower *LINE-1* methylation levels compared to hepatocyte lines (mean 37.52 ± 3.12 vs. 50.73 ± 0.02, *p* = 0.005, Fig 1). *LINE-1* methylation levels were then examined in 40 primary HCC tissues and the corresponding adjacent peritumoral tissues (n = 35). The DNA methylation levels in the HCC primary tissues were significantly lower compared to the adjacent peritumoral tissues (mean 46.45 ± 12.61 vs. 56.09 ± 4.96, *t*-test *p*<0.0001. Fig 2). *LINE-1* DNA methylation levels in healthy livers (mean 57.06 ± 1.7, n = 5) were significantly higher than HCC primary tumors (*p*<0.0001) but were not significantly different from the levels in peritumoral adjacent tissues. *LINE-1* DNA methylation levels were not significantly different between older and younger HCC patients (*p* = 0.24, Table 1) indicating that age-related effects have no major influence in the aberrations of *LINE-1* DNA methylation in HCC.

**Table 1:** Clinicopathological variables of HCC patients and comparison of *LINE-1*, *RASSF1A*, and *CCND2* DNA methylation levels.

HCC n = 40		n	*LINE-1* methylation		*RASSF1A* methylation		*CCND2* methylation	
Age			Mean±SEM	*p* value	Mean±SEM	*p* value	Mean±SEM	*p* value
	<50	12	50.06 ± 3.59	0.24	35.51 ± 7.35	0.48	18.73 ± 4.9	0.7
	>50	28	44.88 ± 2.37		41.69 ± 4.56		20.86 ± 2.37	
**Sex**								
	Male	33	47.94 ± 1.96	0.24	38.90 ± 11.78	0.93	16.73 ± 5.04	0.47
	Female	7	39.35 ± 6.38		40.03 ± 4.05		20.96 ± 2.44	
**Etiology**								
	HBV	7	47.12 ± 5.95	0.79	47.74 ± 7.88	0.37	25.06 ± 7.14	0.26
	HCV	4	41.25 ± 7.12		49.52 ± 10.8		31.48 ± 10.12	
	No infection	29	46.99 ± 2.21		36.59 ± 4.7		17.5 ± 1.98	
**Tumor differentiation**								
	Good	15	49.91 ± 3.05	0.46	28.04 ± 6.28	0.049	16.61 ± 2.62	0.44
	Moderate	17	42.98 ± 3.26		48.63 ± 5.5		24.51 ± 3.97	
	Poor	8	48.12 ± 3.84		40.7 ± 7.6		16.89 ± 3.72	
**Tumor size**								
	<5cm	12	54.99 ± 2.22	0.0007	22.62 ± 6.19	0.003	19.09 ± 3.450 N = 16	0.8
	>5cm	28	42.77 ± 2.38		47.21 ± 4.154		20.62 ± 2.48	
**Stage**								
	I	5	47.71 ± 3.38	0.6156	36.28 ± 5.94	0.45	19.09 ± 3.45	0.68
	II	11						
	III	16	45.58 ± 2.49		42.20 ± 5.10		20.98 ± 2.87	
	IV	8						
**Number of nodules**								
	Uninodular	14	42.94 ± 3.45	0.21	38.46 ± 6.34	0.79	17.31 ± 3.69	0.34
	Multinodular	26	48.32 ± 2.40		40.57 ± 4.93		21.79 ± 2.71	
**Cirrhosis**								
	With Cirrhosis	32	47.53 ± 2.39	0.45	42.51 ± 4.21	0.38	21.37 ± 2.63	0.48
	Without Cirrhosis	8	44.17 ± 3.65		34.29 ± 8.06		17.84 ± 3.99	
**Survival**								
	<3 years	22	44.27 ± 2.45	0.29	40.36 ± 5.09	0.93	18.91 ± 2.10	0.53
	> 3years	18	48.72 ± 3.37		39.62 ± 6.32		21.89 ± 4.24	

### Aberrations of *RASSF1A/CCND2* DNA methylation

**Fig 3:**
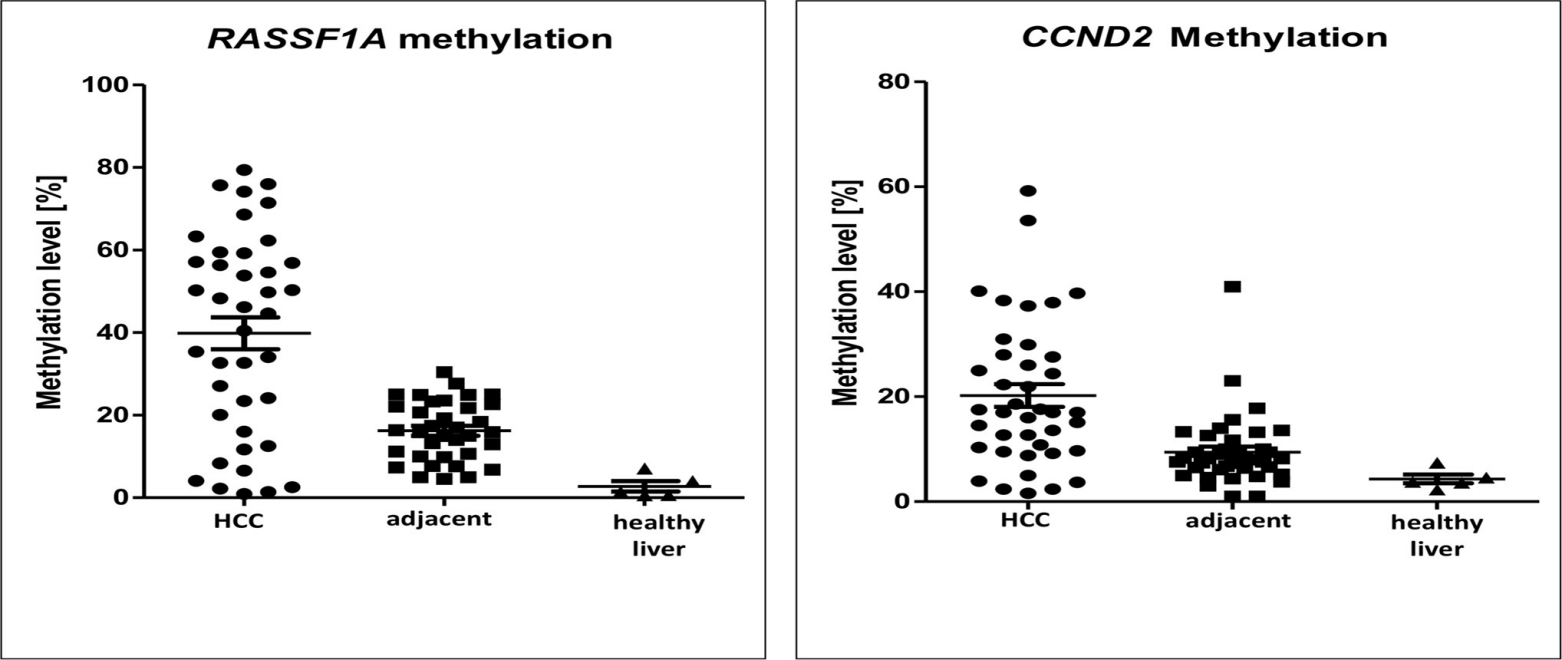
*RASSF1A* and *CCND2* DNA methylation levels in HCC, adjacent peritumoral tissues, and healthy liver. DNA methylation levels were significantly higher in HCC compared to pertitumoral tissues both for *RASSF1A* (mean methylation levels were 39.8 and 16.2, *p*<0.0001, respectively) and *CCND2* (mean methylation levels were 20.2 and 9.4, *p*<0.0001, respectively). For DNA methylation levels of individual CpG sites see S4 Table.

To compare *LINE-1* hypomethylation with gene promoter specific methylation, we quantified DNA methylation levels in *RASSF1A* and *CCND2*, tumor suppressor genes that are frequently methylated in HCC and are associated with the CpG island methylation phenotype (CIMP) [26–28]. We found that *RASSF1A* and *CCND2* DNA methylation levels in HCC primary tissues was significantly higher than in the adjacent peritumoral tissues (39.8±24.3 vs. 16.21±7.2, *p*<0.0001 and 20.22±2.18 vs. 9.43±1.06, *p*<0.0001, respectively, see Fig 3). Using the definition of hypermethylation as explained in the Materials and Methods section (mean methylation + 2xSD), we found frequent *RASSF1* and *CCND2* hypermethylation in HCCs (67.5% and 40%, respectively).

### DNA methylation profiles in benign liver tumors

**Fig 4:**
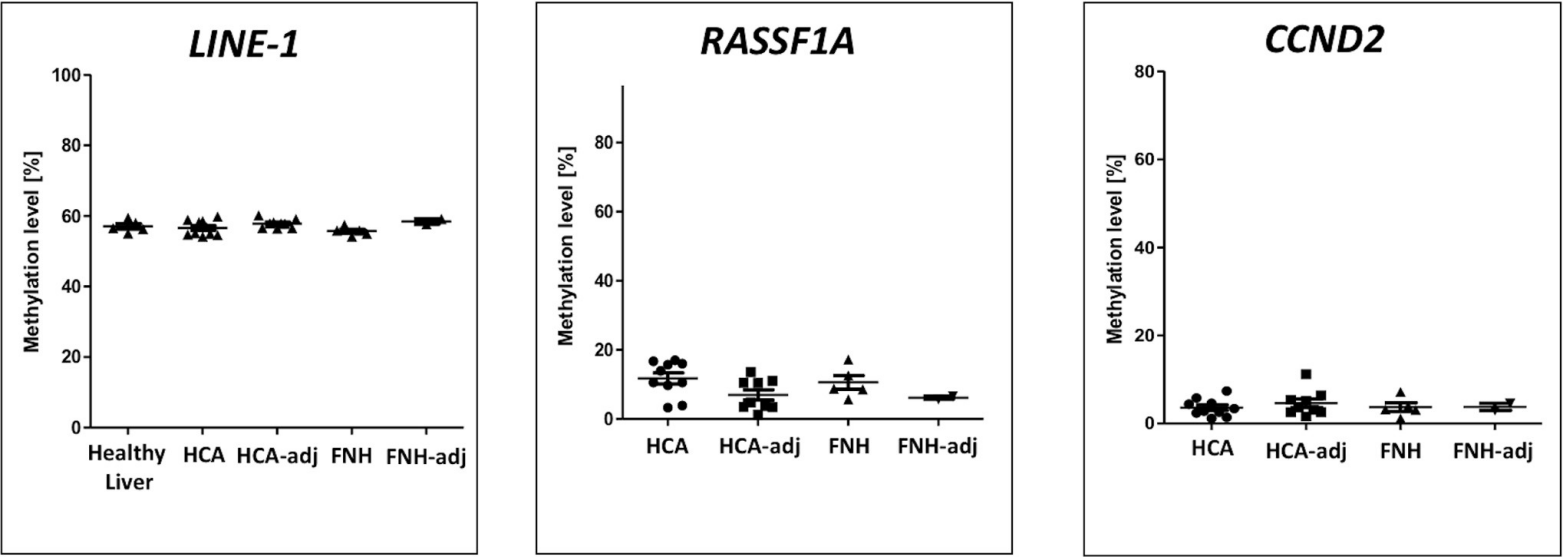
*LINE-1*, *RASSF1A*, and *CCND2* DNA methylation in benign liver tumors (HCA and FNH) and the adjacent peritumoral tissues. DNA methylation levels at the *LINE-1*, *RASSF1A*, and *CCND2* loci were not significantly different in benign liver tumors and the peritumoral tissues. For DNA methylation levels of individual CpG sites see S5 Table.

We then examined *LINE-1* DNA methylation levels in 10 HCAs and 5 FNHs and the corresponding adjacent peritumoral tissues. The *LINE-1* DNA methylation levels in the HCA and FNH primary tissues compared to the adjacent peritumoral tissues were not significantly different (55.65 ± 2.12 vs. 57.82 ± 1.25, *t*-test = 0.14 and 55.65 ± 1.09 vs. 56.5 ± 0.36, respectively, Fig 4). *LINE-1* DNA methylation levels were not significantly different between healthy liver tissues and benign liver tumors (HCA, *p* = 0.64 and FNH, *p* = 0.18). *RASSF1A* and *CCND2* DNA methylation levels were also not significantly different between tumors and the adjacent peritumoral tissues (Fig 4). Hypermethylation was also not detected in HCAs and FNHs.

### Correlation of aberrant *LINE-1* and *RASSF1A/CCND2* methylation with clinicopathological variables

**Fig 5:**
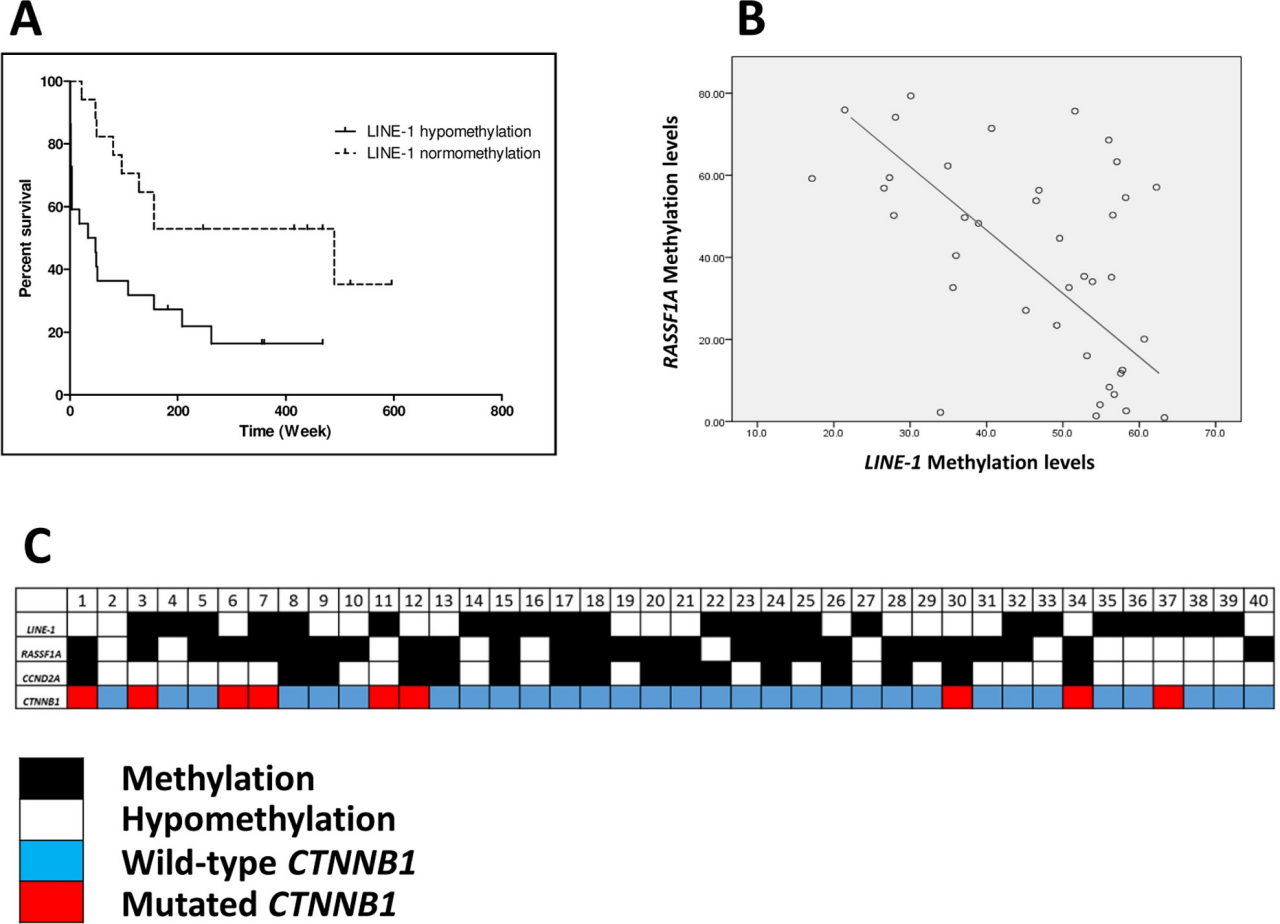
A) Correlation of *LINE-1* hypomethylation and poor survival in HCC. In compared to without methylation changes, *LINE-1* hypomethylation was significantly correlated with shorter HCC survival (median survival 41 and 490 weeks respectively, log rank Mantel-Cox test *p* = 0.007), B) significant inverse correlation between *LINE-1* and *RASSF1* DNA methylation levels in HCC (Spearman r^2^ = -0.47, *p* = 0.002). **C)** Concordant hypermethylation of *RASSF1A* and *CCND2* was associated with *LINE-1* hypomethylation (Fisher exact test *p* = 0.02) but was not correlated with *CTNNB1* positive mutation. Black box represents hypermethylation, white box represents hypomethylation, blue box represents *CTNNB1* wild type, and red box represent *CTNNB1* mutation.

HCC patients with loss of *LINE-1* methylation had significant shorter overall survival (median survival 41 vs. 490 weeks, log rank Mantel-Cox test, *p* = 0.007, see Fig 5A). Other correlations of *LINE-1*, *RASSF1A*, and *CCND2* DNA methylation levels with various clinicopathological parameters are presented in Table 1. Tumor size correlated with lower *LINE-1* DNA methylation and higher *RASSF1A* DNA methylation. Patient age (younger vs. older than 50 years old) did not correlate with *RASSF1A* and *CCND2* DNA methylation levels. In addition, we found an inverse correlation between *RASSF1A* and *LINE-1* DNA methylation levels in HCC (Spearman r^2^ = -0.47, *p* = 0.002, Fig 5B). Hypermethylation of *RASSF1A* and *CCND2* were not associated with shorter overall survival (log rank Mantel-Cox test, *p* = 0.18 and 0.22, respectively). If concurrent *RASSF1A* and *CCND2* hypermethylation is considered as a marker for CIMP-positivity, *LINE-1* hypomethylation was associated with CIMP-positivity (Fisher’s exact test *p* = 0.02).

### *CTNNB1/*β-catenin mutations and the correlation with *LINE-1* hypomethylation as well as *RASSF1A* and *CCND2* hypermethylation

As previous study showed association of *CTNNB1* (β-catenin) gene mutations and HCC with CIMP [29], β-catenin mutation was analyzed in our cohort and revealed that 22.5% (9/40) of HCCs harbored activating mutations (see Fig 5). However, frequency of *CTNNB1* mutations was not significantly correlated with *LINE-1* hypomethylation as well as *RASSF1A* and *CCND2* hypermethylation (Fisher exact test *p* = 0.45 and *p* = 0.9, respectively). Levels of *LINE-1* and *RASSF1A* DNA methylation were also not significantly different between HCCs with and without *CTNNB1* mutations (t-test, *p* = 0.29 and *p* = 0.45, respectively). *CTNNB1* mutations were also not correlated with CIMP-phenotypes.

## Discussion

In the present study we could demonstrate the association of *LINE-1* hypomethylation as a specific event in liver malignancy with shorter overall survival in HCC patients. Our comprehensive literature search in PubMed (search terms: *L1* or *LINE1* or *LINE-1* AND liver AND methylation AND hepatocellular) revealed in total 11 studies addressing *LINE-1* methylation in primary liver tissues [17, 23–25, 30–36] and 3 studies addressing *LINE-1* methylation in the blood of HCC patients [37–39]. Various methods were used for the DNA methylation analysis including MS-PCR [36], Methyl-Light and COBRA [30], quantitative real-time PCR and bisulfite sequencing [33], pyrosequencing [17, 23–25, 34], and array-based genome-wide assessment [35]. Due to the widespread presence of DNA methylation in *LINE-1* sequences under physiological conditions, detection of DNA methylation aberrations requires (semi)-quantitative methods to accurately detect changes specific for the malignant process [40].

Malouf et al. [34] demonstrated significant loss of *LINE-1* methylation in shorter recurrence free- and overall survival of fibrolamellar carcinoma of the liver (FLC) indicating that *LINE-1* hypomethylation might also be a clinically relevant biomarker in closely related HCC samples. Three studies showed that the level of *LINE-1* hypomethylation detected in the peripheral blood correlates with increased risk of HCC [37–39]. This interesting association was also reported in different types of cancer as shown in the meta-analysis by Woo et al. [41]. Therefore, *LINE-1* hypomethylation in white blood cells is commonly found in cancer patients compared to pre-malignant or healthy individuals. Our previous report described concordance of severe loss of DNA methylation at imprinted loci with *LINE-1* hypomethylation in HCC although the biological mechanisms leading to this phenomenon are unknown [17]. Existing studies using genome-wide methylation analysis in HCC [35, 42–45] did not address and analyze specifically the correlation of *LINE-1* methylation with clinical outcome.

Only 4 studies have previously reported significant correlation of *LINE-1* hypomethylation in HCC specimens with unfavorable clinical outcome [23, 24, 25, 33] (in total involving 686 HCC patients with Asian origin), the other 7 studies showed lower *LINE-1* methylation in HCC compared to peritumoral or cirrhotic tissues [17, 30–32, 34–36]. Two of these four studies [24, 25] are from the same group, most likely using an identical patient cohort. In contrast to those previously existing reports, our study analyzed a Caucasian patient cohort that might have different risk factors showing much less association with HBV and HCV infections (Table 1). Despite a decline in HCV infection in the USA a steady increase in HCC incidence is observed [46] highlighting the importance of other risk factors, namely metabolic syndrome, diabetes, chronic alcohol consumption, and non-alcoholic fatty liver disease (NAFLD) [47, 48]. In addition, we used more stringent and quantitative methods to determine hypo/hyper-methylation (mean methylation in healthy liver +/– 2xSD, see Material and Methods section). Previous studies used dichotomous quartiles to determine hypomethylation which might completely depend on the characteristics of individual patient cohorts [24, 25]. Although a generally accepted consensus to determine the threshold for *LINE-1* hypomethylation does not exist, our approach is more objective and universal. In these two previous studies [24, 25], benign liver tumors (HCA, FNH) were not analyzed. Due to the much lower incidence of HCC in the Caucasian population the present study is smaller than the cited studies from East Asia.

The concept of CIMP and its application as an independent prognostic marker in HCC has emerged as an important area in cancer epigenetics [49, 50]. Two tumor suppressor genes, *RASSF1A* and *CCND2*, are the genes most frequently included in HCC-associated CIMP panels [26–28]. Ras signaling pathway activation is found in almost all HCC cases in which epigenetic silencing of Ras and downstream Ras effectors play an important role in liver carcinogenesis [51]. We found that DNA methylation levels of *LINE-1* were negatively correlated with *RASSF1A* gene methylation (Fig 5B). In addition, *LINE-1* hypomethylation is significantly associated with CIMP phenotype as determined by concurrent hypermethylation of *RASSF1* and *CCND2* genes. In contrast to our results, *LINE-1* hypomethylation correlates with the absence of CIMP and microsatellite-stability in colorectal cancer [52, 53]. Future studies need to address the association of *LINE-1* hypomethylation with microsatellite instability, chromosomal instability and CIMP in HCC. Nishida *et al*. [29] have shown that CIMP in HCC is associated with β-catenin (*CTNNB1*) mutations. However, both *LINE-1* hypomethylation and CIMP-phenotypes were not correlated with *CTNNB1* mutations in our Caucasian patient cohort. Although β-catenin activation (through *CTNNB1* mutations) and DNA methylation aberrations represent the most common genetic and epigenetic alterations in liver carcinogenesis, the connection of those alterations are not yet clear [29].

Our present study has demonstrated *LINE-1* hypomethylation as a specific alteration in HCC and its correlation with shorter overall survival. The inverse correlation of *LINE-1* hypomethylation and *RASSF1A* hypermethylation and its association with concurrent *RASSF1A* and *CCND2* hypermethylation indicate the connection with HCC CIMP phenotype. However, future studies using larger Caucasian HCC patient cohorts are required to confirm our results as well as the potential use of *LINE-1* hypomethylation as a predictive marker of therapeutic responses.

## Material and methods

### Study subject

Primary liver tumor specimens from 40 patients with HCCs, 10 patients with HCAs, and 5 patients with FNHs underwent surgical resection at the Hannover Medical School Germany were snap-frozen following a protocol approved by the institutional ethics committee ("Ethik-Kommission der Medizinischen Hochschule Hannover"). The samples for research purposes were collected from diagnostic “left over” material. The primary tissues were then stored at -80°C before subsequent processing for analysis. Classification of liver tumors as well as grading of hepatocellular carcinoma were based on accepted histopathological standards as described in Lehmann *et al*. [40] and Schlageter *et al*.[54]. Primary samples were verified by an experienced pathologist using H&E staining from the reference sections of the snap frozen samples and were included in the analysis if they contained at least 70% of tumor cells. Clinical and pathological data of the study subjects are presented in the Table 1 for HCC and S1 Table for FNH and HCA. Nine cell lines consisting of 7 HCC cell lines (HLE, HLF, HuH7, HepG2, Hep3B, SNU182, and SNU387) and two immortalized hepatocyte lines (THLE-2 and THLE-3) were included in the *LINE-1* DNA methylation analysis. All cell lines were obtained from the American Tissue Culture Collection (ATCC, Rockville, MD, United States) and cultivated in tissue culture media according to the recommendations provided by ATCC. Genomic fingerprints of all included cell lines were verified regularly using short tandem repeat (STR) analysis following the DSMZ’s protocol (DSMZ, Braunschweig, Germany).

### DNA extraction

Extraction of high molecular weight DNA from the fresh-frozen primary specimens and cell lines was performed by overnight digestion with proteinase K (Merck, Darmstadt, Germany) followed by separation using phenol/chloroform (ROTI Carl Roth GmbH, Karlsruhe, Germany) following standard protocols.

### Bisulfite conversion and methylation analysis

For bisulfite conversion, genomic DNA (1000 ng) was treated with sodium bisulfite using EZ DNA Methylation Kit (Zymo Research, HiSS Diagnostics, Freiburg, Germany) following the manufacturers’ protocol. PCR amplification was performed using approx. 25 ng of the bisulfite modified DNA and Platinum Taq DNA Polymerase (Invitrogen, Frankfurt, Germany). Quantification of DNA methylation levels was performed with pyrosequencing (PyroMark, Qiagen, Hilden, Germany) as described previously [55] using primers listed in the Table 2. For each sample, the DNA methylation level was calculated as the mean of all CpG dinucleotide methylation values within the pyrosequencing assay from two independent runs. For *RASSF1A* 7 individual CpG sites were measured, for *CCND2* 5 individual CpG sites, and for LINE-1 7 individual CpG sites. The Pyro-Q-CpG software (Qiagen, Hilden, Germany) was used to analyze the assay quality and levels of DNA methylation from each individual CpG dinucleotide. “Hypomethylation” and “hypermethylation” were defined as methylation value below or above the mean of the methylation level in healthy liver tissues minus or plus two times the standard deviation (Mean_<HL>_ ± 2 × StD), respectively.

**Table 2:** Primers used for pyrosequencing.

PRIMER	Forward	Reverse	Ta (°C)	MgCl2 (mM)	Sequencing
*LINE-1*	TTTTGAGTTAGGTGTGGGATATA	tail-AAAATCAAAAAATTCCCTTTC	60	1.5	AGTTAGGTGTGGGATATAGT
*RASSF1A*	AGTTTGGATTTTGGGGGAGG	tail-CAACTCAATAAACTCAAACTCCCC	58	1.5	GGGTTYGTTTTGTGGTTT
*CCND2*	GTATTTTTTGTAAAGATAGTTTTGATT	tail-CCAAACTTTCTCCCTAAAAAC	55	1.5	CCAAACTTTCTCCCTAAAAAC

### *CTNNB1* mutation detection

Detection of *CTNNB1* mutation was performed in primary HCC samples using primers and protocols as described by Huss *et al*. [56]. Genomic DNA (25ng) was amplified using Platinum II *Taq* Hot-Start DNA polymerase (Invitrogen, Germany) and then was sequenced using GenomeLab DTCS Quick Start kit (Beckman Coulter, Krefeld, Germany) and GenomeLab Genetic Analysis System (Beckman Coulter, Brea, CA) following the manufacturer’s instructions.

### Statistical analysis

GraphPad Prism (version 5.01 for Windows, La Jolla, CA, United States) was used for statistical analysis. Continuous variables of methylation levels in relation with clinicopathological data were compared using the Mann-Whitney-*U* tests. In addition, categorical variables were compared using *χ*^*2*^ tests. Overall survival of HCC patients with different methylation status was compared using Kaplan-Meier curve and long-rank (Mantel-Cox) test. For all comparisons, *p* < 0.05 was considered as statistically significant.

## Supporting information

S1 Table Clinical variables of patients with benign liver tumors, human hepatocellular adenoma (HCA) and focal nodular hyperplasia (HCA). (DOCX)

S2 Table DNA methylation level of individual CpG sites of all samples displayed in Fig 1 (cell lines and healthy liver). (XLSX)

S3 Table DNA methylation level of individual CpG sites of all samples displayed in Fig 2 (HCC, adjacent normal, and unrelated healthy liver tissue). (XLSX)

S4 Table DNA methylation level of individual CpG sites of all samples displayed in Fig 3 (HCC, adjacent normal, and unrelated healthy liver tissue). (XLSX)

S5 Table DNA methylation level of individual CpG sites of all samples displayed in Fig 4 (HCA and adjacent normal, FNH and adjacent normal, and unrelated healthy liver tissue). (XLSX)

## Abbreviations

CIMP: CpG island methylator phenotype
COBRA: Combined bisulfite restriction analysis
DSZM: Deutsche Sammlung für Zellen und Mikroorganismen
FLC: fibrolamellar carcinoma
FNH: focal nodular hyperplasia
HCV: Hepatitis-C virus
HBV: Hepatitis-B virus
HCC: hepatocellular carcinoma
LINE-1: long interspersed nucleotide element 1
MS-PCR: Methylation-specific PCR
STR: short tandem repeat
