# Supplementary material for: LINE-1 hypomethylation in human hepatocellular carcinomas correlates with shorter overall survival and CIMP phenotype
Source: PLoS One. 2019 May 6;14(5):e0216374. doi: 10.1371/journal.pone.0216374 (PMC6502450; doi:10.1371/journal.pone.0216374)
Supplement: S1 Table — (DOCX) [file pone.0216374.s001.docx]

**Supplementary Table S1**. Clinical variables of patients with benign liver tumors, human hepatocellular adenoma (HCA) and focal nodular hyperplasia (HCA)

|  |  | **HCA (n=10)** | **FNH n=5** |
| --- | --- | --- | --- |
| **Age** |  |  |  |
|  | <50y | 10 | 4 |
|  | >50y | 0 | 1 |
| **Sex** |  |  |  |
|  | Male | 1 | 2 |
|  | Female | 9 | 3 |
| **Tumor size** | |  |  |
|  | <5cm | 4 | 4 |
|  | >5cm | 6 | 1 |
| **Number of nodules** | |  |  |
|  | Uninodular | 9 | 5 |
|  | Multinodular | 1 | 1 |
